# Metal-assisted chemical etching of Ge(100) surfaces in water toward nanoscale patterning

**DOI:** 10.1186/1556-276X-8-151

**Published:** 2013-04-02

**Authors:** Tatsuya Kawase, Atsushi Mura, Katsuya Dei, Keisuke Nishitani, Kentaro Kawai, Junichi Uchikoshi, Mizuho Morita, Kenta Arima

**Affiliations:** 1 Department of Precision Science and Technology, Graduate School of Engineering, Osaka University, 2-1, Yamada-oka, Suita, Osaka, 565-0871, Japan

**Keywords:** Dissolved oxygen, Machining, Catalyst, Lithography, Oxygen reduction, Atomic force microscopy

## Abstract

We propose the metal-assisted chemical etching of Ge surfaces in water mediated by dissolved oxygen molecules (O_2_). First, we demonstrate that Ge surfaces around deposited metallic particles (Ag and Pt) are preferentially etched in water. When a Ge(100) surface is used, most etch pits are in the shape of inverted pyramids. The mechanism of this anisotropic etching is proposed to be the enhanced formation of soluble oxide (GeO_2_) around metals by the catalytic activity of metallic particles, reducing dissolved O_2_ in water to H_2_O molecules. Secondly, we apply this metal-assisted chemical etching to the nanoscale patterning of Ge in water using a cantilever probe in an atomic force microscopy setup. We investigate the dependences of probe material, dissolved oxygen concentration, and pressing force in water on the etched depth of Ge(100) surfaces. We find that the enhanced etching of Ge surfaces occurs only when both a metal-coated probe and saturated-dissolved-oxygen water are used. In this study, we present the possibility of a novel lithography method for Ge in which neither chemical solutions nor resist resins are needed.

## Background

Germanium (Ge) is considered to be a substitute for Si for future complementary metal-insulator-semiconductor devices because of its higher carrier mobility than silicon (Si) [1]. Although wet-chemical treatments are essential for the fabrication of Ge-based devices, they have not been well established yet. The primary reason for this is the chemical reactivity of Ge and its oxide (GeO_2_) with various solutions. For example, Ge oxide (GeO_2_) is permeable and soluble in water, unlike the more familiar silicon oxide (SiO_2_). Ge surfaces are also not resistant to various chemical solutions. For example, a piranha solution (a mixture of H_2_SO_4_ and H_2_O_2_) is commonly used in removing metallic and organic contaminants on the Si surface. However, we cannot use it for Ge because it damages Ge surfaces very easily. Although in several earlier works, the etching property of Ge surfaces has been investigated [2,3], the unique chemical nature of Ge prevents researchers from developing surface treatment procedures for Ge using solutions.

One of the surface preparation steps needed is wet cleaning. For Si, sophisticated cleaning procedures have been developed since the 1970s [4,5]. For Ge, however, researchers have just started developing wet cleaning processes together with some pioneering works [6-9]. Furthermore, a variety of solutions have been used in lithography processes (e.g., development, etching, and stripping) to fabricate Si-based devices. However, patterning techniques are not well optimized in the case of Ge. To realize these surface preparation methods, the impact of various aqueous solutions on the morphology of Ge surfaces should be understood on the atomic scale.

In this study, we pay attention to the interaction of water with Ge surfaces in the presence of metals on the Ge surface. In the case of Si, a metal/Si interface in HF solution with oxidants added has been extensively studied [10-18]. Metallic particles on Si serve as a catalyst for the formation of porous surfaces, which can be applied in solar cells. A similar metal/Si interaction is also used to form either oxide patterns or trenches [19]. Recently, we have found that similar reactions occur on Ge surfaces even in water [20,21]. On the basis of these preceding works, we show the formation of inverted pyramids in water on Ge(100) loaded with metallic particles in this study. We also discuss the mechanism of such formation on the basis of the relationship of redox potential as well as the catalytic role of metals. Then, we apply this metal-assisted chemical etching to the nanoscale patterning of Ge in water.

## Methods

We used both p-type and n-type Ge(100) wafers with resistivities of 0.1 to 12 Ω cm and 0.1 to 0.5 Ω cm, respectively. The wafers were first rinsed with water for 1 min followed by treatment with an ultraviolet ozone generator for 15 min to remove organic contaminants. They were then immersed in a dilute HF solution (approximately 0.5%) for 1 min.

We conducted two experiments. One is the etch-pit formation by metallic particles in water. Here, we used both Ag and Pt nanoparticles. Ag nanoparticles with a diameter (φ) of approximately 20 nm were mainly used. To deposit these nanoparticles, Ge surfaces were dipped in HCl solution (10^-3^ M, 100 ml) with AgClO_4_ (10^-4^ M, 100 ml) for 5 min. After dipping, they were dried under N_2_ flow. We also used Pt nanoparticles of approximately 7 nm φ, which were synthesized in accordance with the literature [22]. They were coated with a ligand (tetradecyltrimethylammonium) to avoid aggregation and were dispersed in water. This enabled us to obtain near monodispersed particles. The Ge samples were immersed in the resulting solution and dried under N_2_ flow. Then, the Ge surfaces loaded with the Pt particles were treated with the ultraviolet ozone generator for 6 h to remove the ligand bound to the Pt surfaces. In this experiment, we used two types of water with different dissolved oxygen concentrations, both of which were prepared from semiconductor-grade ultrapure water. The first type was water poured and stored in a perfluoroalkoxy (PFA) beaker. This water has a saturated dissolved-oxygen concentration of approximately 9 ppm. The second type contained a very low oxygen concentration of approximately 3 ppb. We, hereafter, call these two types of water ‘saturated dissolved-oxygen water’ (SOW) and ‘low dissolved-oxygen water’ (LOW), respectively. By putting a Ge sample in a PFA container connected directly to an ultrapure water line faucet, we were able to treat samples in LOW. The change in the structure of Ge surfaces loaded with metallic particles by immersion in water in the dark was analyzed by scanning electron microscopy (SEM, HITACHI S-4800, Hitachi Ltd., Tokyo, Japan).

The other experiment is the nanoscale machining of Ge surfaces by means of the catalytic activity of the metallic probes, using a commercial atomic force microscopy (AFM) system (SPA-400, Hitachi High-Tech Science Corporation, Tokyo, Japan) equipped with a liquid cell. It was carried out in the contact mode using two types of silicon cantilever probe from NANOWORLD (Neuchâtel, Switzerland): a bare Si cantilever and a cantilever coated with a 25-nm thick Pt/Ir layer (Pt 95%, Ir 5%). The resonant frequency and spring constant of both probes were 13 kHz and 0.2 N/m, respectively. An AFM head was covered with a box capable of shutting out external light. A conventional optical lever technique was used to detect the position of the cantilever. Ultrapure water exposed to air ambient and poured in the liquid cell contained approximately 9 ppm dissolved oxygen (SOW). We added ammonium sulfite monohydrate (JIS First Grade, NACALAI TESQUE Inc., Kyoto, Japan) to the water in the liquid cell. Performed according to the literature [23-25], this method enabled us to obtain ultralow dissolved-oxygen water with approximately 1 ppb oxygen (LOW).

## Results and discussion

**Figure 1:**
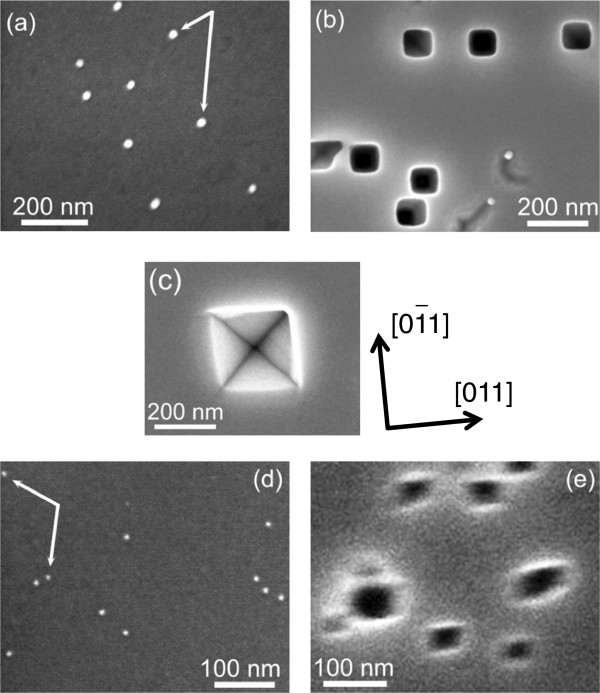
**SEM images of a p-type Ge(100) surface loaded with metallic particles.** (**a**) After deposition of Ag particles (φ 20 nm). (**b**) After immersion in water for 24 h. (**c**) After immersion in water for 72 h. Crystallographic directions are given for this figure, indicating that the edges of the pits run along the <110> direction. (**d**) After deposition of Pt particles (φ 7 nm). (**e**) After immersion into water for 24 h. Square pits, probably representing inverted pyramids, are formed as well as some pits with irregular shapes such as ‘rhombus’ and ‘rectangle’. In (**a**) and (**d**), some particles are indicated by white arrows. In (**b**), (**c**), and (**e**), the samples were immersed in saturated dissolved-oxygen water in the dark.

Figure 1a shows a typical p-type Ge(100) surface after the deposition of Ag particles. From the figure, it is clear that the particles are well dispersed (not segregated) and almost spherical, even with the simple deposition method used. They are approximately 20 nm in diameter. After the sample was immersed and stored in SOW in the dark for 24 h, its surface structure changed markedly, as shown in Figure 1b. Namely, most of the Ag particles disappeared and pits emerged. Most of the pits formed square edges. When the sample was dipped in SOW for more 48 h (72 h in total), each pit grew as shown in Figure 1c. It is clear that the shape of the pit is an inverted pyramid with edges aligned along the <110> direction. We confirmed in another experiment that (1) a metallic particle usually resided at the bottom of the pit [21], and (2) inverted pyramidal pits were formed on the n-type Ge sample as well. Figure 1d shows an SEM image of a p-type Ge(100) surface loaded with Pt particles. As indicated by white arrows, particles of about 7 nm φ are well dispersed. After the surface shown in Figure 1d was subsequently immersed in SOW and stored in the dark for 24 h, etch pits were formed as shown in Figure 1e.

Many works have shown pore formation on Si with metallic particles as catalysts in HF solution containing oxidants such as H_2_O_2_[10-18]. In analogy with these preceding works, it is likely that an enhanced electron transfer from Ge to O_2_ around metallic particles is the reason for the etch-pit formation shown in Figure 1b,c,e. The reaction by which O_2_ in water is reduced to water can be expressed by the redox reaction equation:

(1) $$\begin{array}{c} O_{2}+4H^{+}+4e^{-}↔2H_{2}O,\\ E_{0}=+1.23\;\left(V\;\mathrm{vs}.\;\mathrm{NHE}\right), \end{array}$$

where *E*_0_ is the standard reduction potential, and NHE is the normal hydrogen electrode. The reaction in which Ge in an aqueous solution releases electrons can be expressed as

(2) $$\begin{array}{c} {\mathrm{GeO}}_{2}+4H^{+}+4e^{-}↔\mathrm{Ge}+2H_{2}O,\\ E_{0}=-0.15\;\left(V\;\mathrm{vs}.\;\mathrm{NHE}\right). \end{array}$$

**Figure 2:**
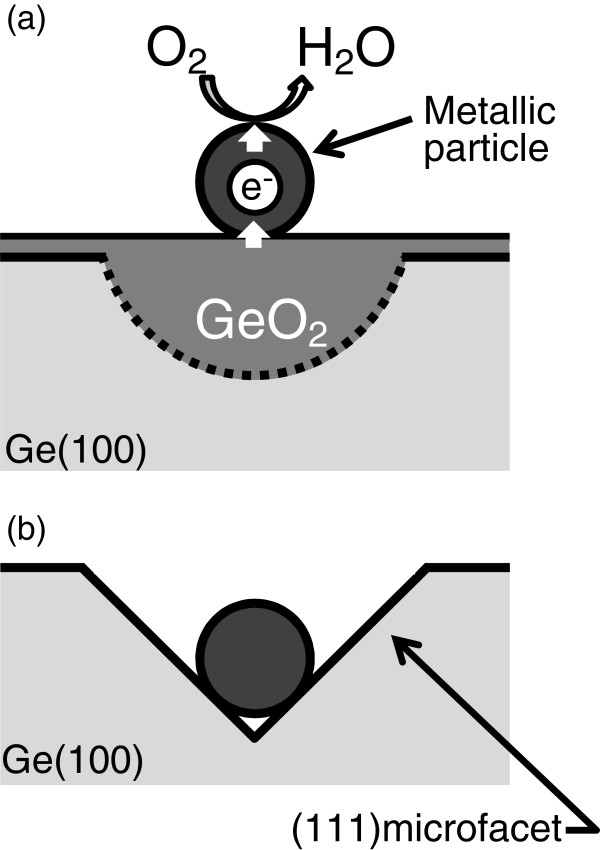
**Schematic depiction of metal-induced pit formation in water.** (**a**) Owing to the catalytic activity of metallic particles, electrons are transferred from the Ge bulk to water to reduce dissolved O_2_ to H_2_O molecules, which leads to an enhanced oxidation around the particles. (**b**) GeO_2_ dissolves in water, leaving (111) microfacets.

Because the redox potentials depend on the pH of the solution, these potentials at 25°C are respectively given by the Nernst relationship as

(3) $$E_{O_{2}}=+1.23-0.0591\times \mathrm{pH}\left(V\;\mathrm{vs}.\;\mathrm{NHE}\right),$$

(4) $$E_{{\mathrm{GeO}}_{2}}=-0.15-0.0591\times \mathrm{pH}\left(V\;\mathrm{vs}.\;\mathrm{NHE}\right),$$

where the O_2_ pressure is assumed to be 1 atm. In water of pH 7, $E_{O_{2}}$ and $E_{{\mathrm{GeO}}_{2}}$ are +0.82 and -0.56 (V vs. NHE), respectively. These simple approximations imply that a Ge surface is oxidized by the reduction of dissolved oxygen in water. We speculate that such oxygen reduction is catalyzed by metallic particles such as Ag and Pt. Electrons transferred from Ag particles to O_2_ in water are supplied from Ge, which enhance the oxidation around particles on Ge surfaces, as schematically depicted in Figure 2a. Because GeO_2_ is soluble in water, etch pits are formed around metallic particles, as shown in Figure 1. We showed in another experiment that the immersion of a Ge(100) sample loaded with metallic particles (Ag particles) in LOW creates no such pits [20,21], which gives evidence of the validity of our model mentioned above. Furthermore, we have confirmed that the metal-assisted etching of the Ge surfaces in water mediated by dissolved oxygen occurs not only with metallic particles but also with metallic thin films such as Pt-Pd [20] and Pt [21].

One may wonder why p-type Ge releases electrons to be oxidized as shown in Equation (2), because electrons are minority carriers for p-type samples. In the pore formation on Si by metal-assisted chemical etching in the dark, researchers mentioned that the conductivity type of the Si substrate (p-type or n-type) does not directly influence the morphology of pits formed [11,12]. This is in agreement with our result in which a Ge surface with either conductivity type was preferentially etched around metallic particles in saturated dissolved-oxygen water in the dark. As described previously, we confirmed that similar etch pits to those on p-type wafers were formed on n-type ones. We presume that n-type Ge samples emit electrons in the conduction band (majority carriers), whereas p-type samples release them in the valence band.

In our experiments, most etch pits were pyramidal, one of which is shown in Figure 1c. The outermost Ge atoms on the (111) and (100) faces have three and two backbonds, respectively. This probably induces a (100) facet to dissolve faster in water than a (111) facet, forming a pyramidal etch pit on the Ge(100) surface, as schematically shown in Figure 2b. This anisotropic etching is very unique, because it has not been observed on Si(100) surfaces with metallic particles immersed in HF solution with oxidants. It should be noted that Figure 1e exhibits some ‘rhomboid’ and ‘rectangular’ pits together with ‘square’ pits. We believe that the square pits in Figure 1e represent pyramidal etch pits similar to those with Ag particles in Figure 1c. On the other hand, the reason for the formation of the rhomboid or rectangular pits in Figure 1e is not very clear at present. It is possible that the shape of a pit depends on that of a metallic particle. Although Ag particles (φ is approximately 20 nm) appear spherical in Figure 1a, the shape of the Pt particles (φ about 7 nm) is hard to determine from the SEM image in Figure 1d. To answer this question, etch pits should be formed with Ag and Pt particles of similar diameters and shapes, which remain to be tested.

**Figure 3:**
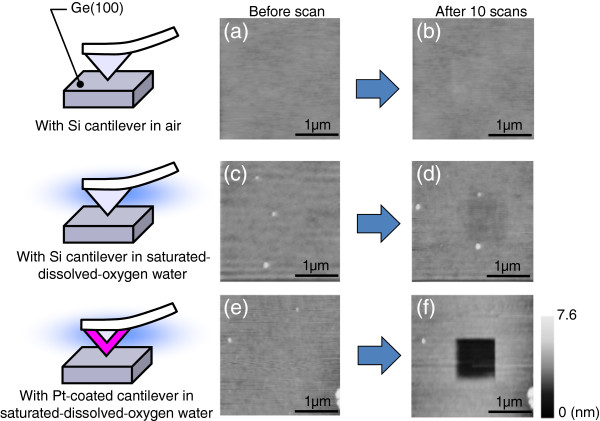
**AFM images to demonstrate metal-assisted patterning of Ge(100) surfaces in water.** In the left column, experimental conditions are schematically depicted. (**a**), (**c**), (**e**) are the initial Ge surfaces before scans. (**b**) Image after ten scans of 1.0 × 1.0 μm^2^ central area in air with Si cantilever. (**d**) Image after scans in saturated dissolved-oxygen water (SOW) with Si cantilever. (**f**) Image after ten scans in SOW with Pt-coated cantilever. In (**b**), (**d**), and (**f**), the pressing force was set to 3 nN.

**Figure 4:**
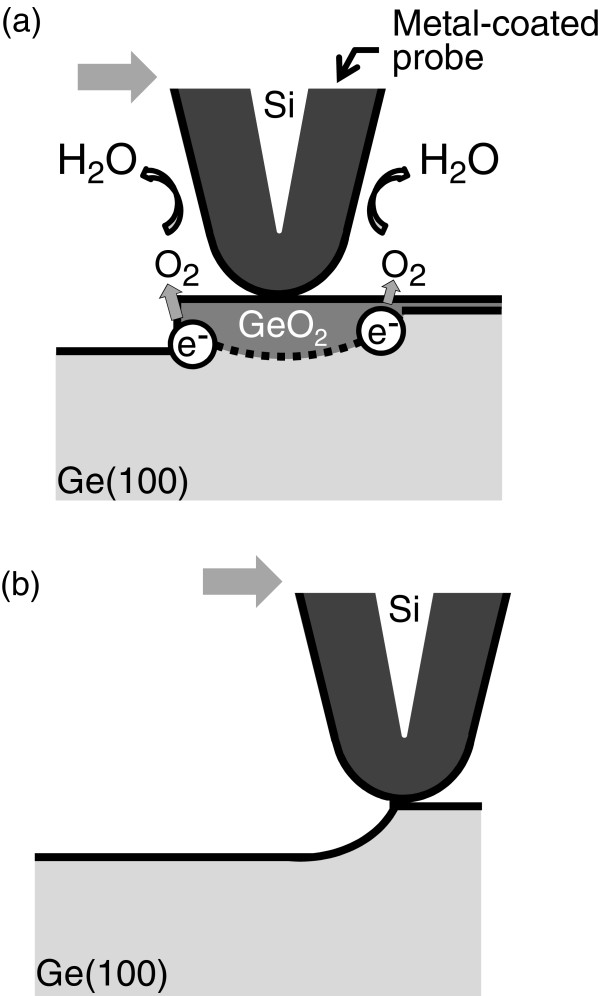
**Schematic depiction of metal-assisted patterning of Ge surfaces in water.** (**a**) Metals coated on a cantilever catalytically oxidize a Ge surface, the mechanism of which is the same as that shown in Figure 2a. (**b**) Surface areas in contact with the metal probe are removed continuously in water during the scans, owing to the soluble nature of GeO_2_.

**Figure 5:**
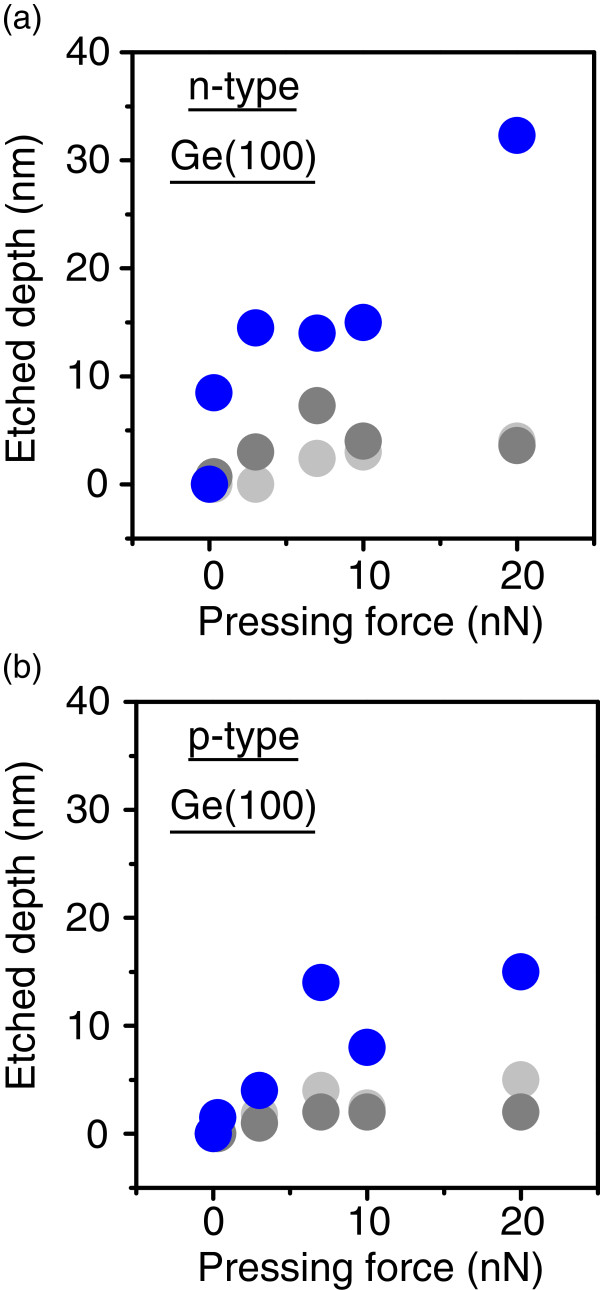
**Etched depth as a function of pressing force.** (**a**) and (**b**) were obtained on n-type and p-type Ge(100) surfaces, respectively. Blue and gray filled circles represent data with Pt-coated cantilevers in saturated dissolved-oxygen water (SOW) and low-dissolved-oxygen (LOW) water, respectively. Light gray filled circles were obtained with a Si cantilever in SOW.

On the basis of the experimental results shown above, we aimed at the nanoscale patterning of Ge surfaces in water by scanning a metal-coated probe. An example is shown in Figure 3 in which experimental conditions are schematically depicted on the left column. First, a p-type Ge(100) surface was imaged using a conventional Si cantilever in air in the contact mode with a scan area of 3.0 × 3.0 μm^2^, as shown in Figure 3a. Then, the 1.0 × 1.0 μm^2^ central area was scanned ten times with a pressing force of 3 nN, and the 3.0 × 3.0 μm^2^ initial area was imaged again. The ten scans took about 45 min. Significant changes in Figure 3a,b are hardly visible, indicating that the mechanical removal of the Ge surfaces by the cantilever is negligible. Experiments similar to those shown in Figure 3a,b were performed in SOW, and their results are shown in Figure 3c,d, respectively. In Figure 3d, the scanned area at the center of the image is observed as a shallow hollow, the cross-sectional profile of which revealed its depth to be approximately 1.0 nm. In contrast, the multiple scans (ten scans) using a Pt-coated cantilever in SOW created a clear square hollow, as shown in Figure 3e,f. The etched depth of the 1.0 × 1.0 μm^2^ central area in Figure 3f was about 4.0 nm from a cross-sectional profile. The mechanism of inducing the difference between image (d) and image (f) in Figure 3 is as follows. As mentioned previously, we scanned a cantilever in the contact mode. Taking into account the catalytic activity of metals (e.g., Pt) enhancing the reactions in Equations (1) and (2), we suppose that, at each moment during the scan, a Ge surface in contact with a Pt probe is preferentially oxidized in water in the presence of dissolved oxygen. This is schematically drawn in Figure 4a. Owing to the soluble nature of GeO_2_, the scanned area exhibits a square hollow, as shown in Figure 3f. In Figure 3b,d,f taken after the ten scans, no pyramidal pits such as those shown in Figure 1 are observed. This is because we did not fix the cantilever at only one surface site, but rather scanned it over a micrometer area, which is much larger than the etched depth, as schematically depicted in Figure 4b. Figure 5a,b shows a summary of etched depth as a function of pressing force on the n-type and p-type Ge(100) surfaces, respectively. Because the plots in Figure 5 slightly fluctuate, it is hard to fit them using a simple straight line or a curve. This is probably due to the difference in probe apex among the sets of experiments performed. However, Figure 5 clearly indicates that (1) the catalytic activity of metals (e.g., Pt) has a much greater effect on Ge etching than that of the mechanical machining caused by a pressurized cantilever, and (2) dissolved oxygen in water is the key molecule in metal-assisted etching. Namely, it is easy to imagine that the Ge surface was machined mechanically to some extent by the pressed cantilever on Ge. In Figure 5, the etched depth increases slightly at a larger pressing force even with a Si cantilever in SOW (light gray filled circles) or a Pt-coated cantilever in LOW (gray filled circles). This indicates that the mechanical etching of Ge occurs, but its effect is very small. On the other hand, a drastic increase in etched depth is observed with a Pt-coated cantilever in SOW (blue filled circles) at each pressing force, which is probably induced by the catalytic effect of Pt mediated by dissolved oxygen in water. One may think that the difference in etched depth between the blue and gray (or light gray) filled circles increases with increasing pressing force in Figure 5. This is as if the catalytic effect is enhanced at greater pressing forces. As for the reason for this enhancement, we imagine that the probe apex became blunter at larger forces. This results in an increase in the area of contact between the metallic probe and the Ge surface, which enhances the etching rate of Ge by the catalytic effect. Note in Figure 5 that an n-type Ge surface is etched deeper than a p-type one in the entire pressing force range when a Pt-coated cantilever was scanned in SOW. One explanation for this is that more electrons in the n-type Ge samples are transferred to oxygen molecules via Equations (1) and (2) because the work function, or the energy necessary for an electron to escape into vacuum from an initial energy at the Fermi level, is smaller for n-type samples than for p-type ones. This increases the oxidation rate of Ge, resulting in an accelerated etching of n-type Ge. Another explanation is that the resistivity of the samples, not the conductivity type, determines the etched depth shown by a blue filled circle in Figure 5. Because our p-type samples had a wider range of resistivities (0.1 to 12 Ω cm) than the n-type ones (0.1 to 0.5 Ω cm), we should not exclude the possibility of carrier density affecting the removal rate of Ge in metal-assisted chemical etching.

As mentioned in the ‘Background’ section, Ge is not resistant to a variety of chemical solutions. Hence, wet-chemical treatments such as wet cleaning and lithography for Ge have not been well optimized compared with those for Si. The results in this study present several important messages for future semiconductor processes for Ge. First, residual metallic particles on Ge can increase surface microroughness even in water. For Ge surfaces, LOW should be used for rinsing to prevent unwanted pit formation. However, the metal-assisted chemical etching presented here can be a novel patterning technique for Ge surfaces in water, one example of which is demonstrated in Figures 3 and 5. This method is unique and promising because it requires no chemical solution that degrades Ge surfaces but is used in conventional wet-chemical treatments in Si processes.

## Conclusions

We studied the metal-induced chemical etching of Ge(100) surfaces in water. We showed that noble metal particles such as Ag and Pt induce anisotropic etching. The mechanism of this formation is the catalytic activity of noble metals to reduce O_2_ molecules in water, which promotes preferential oxidation around metallic particles. Etch pits are formed to roughen the surface due to the soluble nature of GeO_2_. A key parameter for controlling the reaction is the dissolved oxygen concentration of water. We proposed that enhanced etching can be used positively toward the nanoscale patterning of Ge surfaces in water. This idea was confirmed by a set of AFM experiments in which a cantilever probe on Ge(100) was scanned in either water or air. We investigated the dependences of probe material, pressing force, and dissolved oxygen concentration on etched depth. We demonstrated the metal-assisted patterning of Ge surfaces in water, the mechanism of which is similar to that of the metal-induced pit formation mentioned above.

## Abbreviations

AFM: Atomic force microscopy; LOW: Low dissolved-oxygen water; NHE: Normal hydrogen electrode; PFA: Perfluoroalkoxy; SEM: Scanning electron microscopy; SOW: Saturated dissolved-oxygen water

## Competing interests

The authors declare that they have no competing interests.

## Authors' contributions

TK carried out the nanoscale patterning experiments using the AFM setup. AM investigated the etching property of the Ge surface by metallic particles by SEM. KD and KN participated in the sample preparations. KK and JU analyzed the data, and MM revealed the nanoscale mechanism of metal-assisted chemical etching. KA gave the final approval of the version of the manuscript to be published. All authors read and approved the final manuscript.
